# Supplementary material for: Individual dopaminergic neurons induce unique, yet overlapping combinations of behavioural modulations, including safety learning, memory retrieval, and acute locomotion
Source: eLife. 2026 Oct 16;14:RP106148. doi: 10.7554/eLife.106148 (PMC13675010; doi:10.7554/eLife.106148)
Supplement: Peer review history

# Editor report: eLife Assessment

**DOI:** 10.7554/eLife.106148.3.sa0

**Authors:** Meet Zandawala

**Affiliations:** University of Nevada, Reno United States

**Keywords:** Convincing, Valuable

This study presents **valuable** findings on the role of specific dopamine neurons for aversive learning and modulation of innate behavior in *Drosophila* larvae. The authors present **convincing** evidence backed up by detailed behavioral quantification and rigorous testing. Their data confirms previous findings and will be of interest to the learning and memory community.

# Referee report: Reviewer #1 (Public review)

**DOI:** 10.7554/eLife.106148.3.sa1

Summary:

The authors investigate the role of different specific dopaminergic neurons in the mushroom body of *Drosophila* larvae for learning and innate behavior. All the tested neurons are thought to be involved in punishment learning. The authors discover that artificial activation of single DANs in training leads to safety learning, but not punishment learning. Furthermore, activation of single DANs can lead to changes in locomotion behavior, which can affect light preference. The authors provide a deeper understanding of the functional diversity of single dopamine neurons; however, it is unclear how translatable these findings are to learning experiments with real punishment stimuli.

The authors provide a detailed behavioral analysis of locomotion in response to activation of various dopamine neurons. This analysis allows them to exclude that the locomotion defects affect memory recall behavior.

Strengths:

The authors disentangle which kind of memories are formed with the activation of different dopamine neurons - safety learning and/or punishment learning. They further investigate whether the US is required in the test for recall. They do indeed find differences, and the results will be of interest to the learning and memory community.

Interestingly, optogenetic activation of a single DAN during training leads to safety memory, but not punishment memory. Furthermore, DAN activation also affects innate locomotion, and the authors show that optogenetic activation of different DANs affects locomotion differently.

Weaknesses:

All experiments in the manuscript use optogenetic activation of DANs, thus it is not clear what kind of memories are formed. Several stimuli can be used as punishment, such as electric shock, salt, bitter, and light - it is not clear what kind of memory the authors investigate here. The findings could be discussed in the context of what DANs respond to. Furthermore, studies in adults and larvae showed that most DANs can code for both valences - etc., aversive DANs can be activated by punishment, and inhibited by reward. Thus, safety learning might be a result of a decrease in activity in DANs during odor presentation. The authors also do not discuss possible feedback loops from MBONs to DANs across compartments. Could such connections allow for safety learning in larvae?

The authors show that artificial activation with different light intensities can form different memories and that increasing the light intensity sometimes leads to no memories. Also, using different optogenetic tools reveals different results. This again raises the question of how applicable the results will be for learning with real stimuli. Is there a natural stimulus that only induces safety learning, but no punishment learning? The authors discuss these limitations.

# Referee report: Reviewer #2 (Public review)

**DOI:** 10.7554/eLife.106148.3.sa2

Summary:

This study provides valuable context for ongoing research on the role of dopamine in memory and locomotion. DANs have been a fascinating area of study due to their complexity, and this work dissects specific DANs, exploring their roles in different memory-related behaviors while offering some explanations. The discussions provided by the authors effectively situates the study in the broader field of learning, memory, DAN circuitry and behavioral computation in insect brains. The study achieves what it sets out to and it does so unequivocally. The experiments were elegantly designed, leaving little room for doubt in the study's claims. However, the study lacks context regarding the molecular pathways underlying these results. While it strengthens current knowledge by providing robust evidence, it does little to explore the molecular mechanisms behind these effects.

Strengths:

(1) Experiment design is one of the strengths of this study. The experiments are thorough and cover the length and breadth of the core findings of the study. Although a lot of work has already been done in studying the role of dopamine in memory and locomotion, the dissection of the functions of distinct DANs in larvae has been done meticulously with well-structured experiments.

(2) This study fits quite nicely into the puzzle of memory, especially in the context of Dopamine. Previous studies in *Drosophila* adults have shown the opposing roles of DANs in locomotion depending on the context of DAN activation. This study drives that point home for larvae, providing conclusive evidence in that regard.

(3) The use of clear figures and simple language is one of the strengths of this paper. The figures are comprehensive, complete and manage to narrate the story by themselves. The flow of information is smooth. The simple and effective language used maintains scientific rigor while remaining accessible to those new to the field. A pleasant read.

Weaknesses:

(1) The authors have done a great job at structuring the figures. But some main figures would benefit from including the controls instead of placing them in supplementary.

(2) The paper would benefit from a deeper discussion regarding molecular mechanisms underlying their results. It would be interesting to see what the authors think about different Dopamine receptors and how they relate to the findings of this paper.

(3) Throughout the paper, the authors have been clear and comprehensive, but in some cases, further explanation of their choices were missing. For example, the choice to compare bending and tail velocity over other parameters within the same clusters is unclear.

Comments on revised version.

Most of the comments have been addressed.

# Referee report: Reviewer #3 (Public review)

**DOI:** 10.7554/eLife.106148.3.sa3

Summary

Across species, dopamine release serves seemingly diverse functions, such as reinforcing memories and regulating locomotion and flight. However, whether distinct dopaminergic neurons (DANs) are allocated to each function is unclear. In this study, Toshima et al. have used the numerically simple organization of the *Drosophila* larval brain to answer this question. They use optogenetic activation to systematically stimulate a small set of DANs, individually and collectively, and study the effect on diverse functions such as memory formation, retrieval, and locomotion. The reproducibility of optogenetic activation is a strength of this approach. At the same time, this is a caveat, as optogenetic activation may not recapitulate natural modes of activation and may lead to outcomes not observed under natural conditions. They find that singly or collectively, DL1 DANs can induce punishment and/or safety memory formation and retrieval. DANs can even gate the expression of memory. Finally, the same DANs also modulate locomotion in the larvae. The authors speculate that dopaminergic neurons in other species may also share such overlapping functions. Their findings are nicely summarised in Figure 9.

Strengths

The study systematically activates neurons in the DL1 cluster. Individual and collective stimulation of the Dl1 DANs has been conducted to assess the induction and gating of aversive punishment memory, safety memory, and acute locomotion.

Specific adult *Drosophila* DANs are known to induce dual behaviors and functions. The same MP1/y1pedc DANs are recognized for gating appetitive memory expression and representing aversive teaching signals downstream of sensory stimuli such as electric shocks, bitter tastes, and heat. Neurons in the PPL1 cluster regulate adult flight and food-seeking behavior. The authors deserve credit for conducting an organized examination of dopaminergic neuronal functions in larvae, thereby making their findings more comparable and facilitating the proposal of a holistic model.

They have provided substantial evidence for their findings and have frequently presented replicated behavioral datasets. They have been transparent about the results that were difficult to explain. Additionally, they have provided an impressive body of supporting data to strengthen their main findings.

Weaknesses

As mentioned above, optogenetic activation may not recreate natural neuronal activation in response to external stimuli. This could have led to outcomes that will not occur under other natural circumstances.

Comments on revised version.

I appreciate the author's responses, and I do not have any comments or suggestions at this point.

# Author comment: Author response

**DOI:** 10.7554/eLife.106148.3.sa4

**Authors:** Naoko Toshima, Arman Behrad, Franziska Behnke, Gauri Kaushik, Alice Weiglein, Martin Strauch, Juliane Thoener, Oliver Kobler, Maia Lisandra M Wang, Ayaka Fukushima, Markus Dörr, Michael Schleyer

**Affiliations:** Hokkaido University Sapporo Japan; Leibniz Institute for Neurobiology Magdeburg Germany; Leibniz Institute for Neurobiology Magdeburg Germany; Hokkaido University Sapporo Japan; Leibniz Institute for Neurobiology Magdeburg Germany; RWTH Aachen University Aachen Germany; Leibniz Institute for Neurobiology Magdeburg Germany; Leibniz Institute for Neurobiology Magdeburg Germany; Hokkaido University Sapporo Japan; Hokkaido University Sapporo Japan; Leibniz Institute for Neurobiology Magdeburg Germany; Hokkaido University Sapporo Japan

The following is the authors’ response to the original reviews.

**Public Reviews:**

**Reviewer #1 (Public review):**

Weaknesses:

All experiments in the manuscript use optogenetic activation of DANs, thus it is not clear what kind of memories are formed. Several stimuli can be used as punishment, such as electric shock, salt, bitter, and light - it is not clear what kind of memory the authors investigate here. The findings could be discussed in the context of what DANs respond to.

This is indeed a caveat of our study and we discuss this issue now in lines 557-566. We refrained from testing necessity to specific US on purpose as we knew that another research group was focussing on this question in parallel (Weber et al., 2023, also published in eLife) and therefore rather focussed on complementary experiments. That study also includes a rather deep discussion about the inputs to individual DANs. We briefly refer to this discussion (lines 442-444) but decided to not go into detail to avoid too much overlap.

Furthermore, studies in adults and larvae showed that most DANs can code for both valences - etc., aversive DANs can be activated by punishment, and inhibited by reward. Thus, safety learning might be a result of a decrease in activity in DANs during odor presentation. The authors also do not discuss possible feedback loops from MBONs to DANs across compartments. Could such connections allow for safety learning in larvae?

We thank the reviewer for raising these points and included a brief discussion of both scenarios in lines 469-474.

The authors show that artificial activation with different light intensities can form different memories and that increasing the light intensity sometimes leads to no memories. Also, using different optogenetic tools reveals different results. This again raises the question of how applicable the results will be for learning with real stimuli. Is there a natural stimulus that only induces safety learning, but no punishment learning?

We do not know of such a stimulus. Based on our data, a US that only activates a single DAN should only make safety memory – however, the available data of which US activates which DAN is very limited in larvae and currently no such US is known. We discuss this point briefly in lines 557-563 and 572-574.

The authors provide a detailed behavioral analysis of locomotion behavior; however, the detailed analysis seems unnecessary for that dataset. Modulation of speed and bending rate has been described before with simpler methods (specifically for MBONs). The revealed locomotion phenotypes probably affect larval locomotion during memory recall with light activation, thus the authors should show that larvae are potentially able to move during light-on memory tests.

We expanded our locomotion analysis of the innate and learned odor preference experiments (new Fig. 6) and show that in these experiments, even with TH-DANs being activated, larvae indeed can move relatively normal and the existing locomotion phenotypes are not correlated to their olfactory choices.

We do not agree that the locomotion analysis is unnecessary. Modulations of speed and bending have been described for MBONs but to our knowledge not for DANs. It is not trivial at all that DANs and MBONs cause the same behavioral modulations (see, for example, this adult study: Mohammad et al., 2024 Plos Biol). There is extremely limited knowledge about the motoric effects of dopaminergic neurons in larvae - we therefore find it important to describe our results in detail. We added some further rationale of why we think it is crucial to explore the functions of DANs for learning and movements together (lines 81-86).

**Reviewer #2 (Public review):**

Weaknesses:

(1) The authors have done a great job at structuring the figures. But some main figures would benefit from including the controls instead of placing them in supplementary.

We had decided to put the controls into the supplement in some cases to prevent the main figures to be overcrowded. We revised this decision upon the reviewer’s comment for Fig. 8 (previously Fig. 7) but decided to keep other figures unchanged as we feel that the current design best fits the purpose of each figure. We provide a figure-for-figure rationale in our response to the recommendations for the authors.

(2) The paper would benefit from a deeper discussion regarding molecular mechanisms underlying their results. It would be interesting to see what the authors think about different Dopamine receptors and how they relate to the findings of this paper.

We thank the reviewer for the suggestion. Although we agree that such a discussion would be interesting, we hesitate to expand on this topic, as the discussion is already quite long and our study does not contribute any new data to clarify the molecular dopaminergic mechanism.

(3) Throughout the paper, the authors have been clear and comprehensive, but in some cases, further explanation of their choices were missing. For example, the choice to compare bending and tail velocity over other parameters within the same clusters is unclear.

We understand that this choice was not clearly explained and expanded on our rationale in lines 244-251.

**Reviewer #3 (Public review):**

Weaknesses:

The larvae exhibit directed locomotory action to express punishment or safety memory. If the larvae did not move, we would not be able to assess memory function. Hence, functional activation of DANs could result in one action, which seems like two different functions of memory expression and locomotion. It can also be argued that activation of DANs represents a teaching signal to the KCs, and then eventually, downstream of the MBONs, it results in locomotion modulation. Hence, the seeming functional diversity could be a function of different downstream neuronal pathways and not molecular context-dependent diversity inside dopaminergic neurons. The authors should address this possibility or point out the fallacy in the above argument.

We thank the reviewer for raising this issue. To the first point, we expanded our locomotion analysis of the innate and learned odour preference experiments (new Fig. 6) and show that in these experiments, even with TH-DANs being activated, larvae indeed can move relatively normal. In addition, the existing locomotion phenotypes in these experiments were not correlated with the animals’ olfactory choice. This makes it unlikely that the changed locomotion directly determines our observation during the olfactory experiments.

We do agree that it is possible that both the preference after learning and the changed locomotion could come through the same dopaminergic mechanism via diverse downstream pathways. We cover this hypothesis in Fig. 10G and address this question briefly in lines 580-582.

The finding that activation of TH-GAL4 conveys aversive valence and R58E02-GAL4 conveys appetitive valence seems redundant (Figure 6). I understand they say this in the context of locomotion. However, they may not have mentioned similar findings in adults. In adults, artificial activation of DANs covered by the same GAL4 lines acts as aversive and appetitive teaching signals for memory formation. These references should be cited appropriately in the results and discussion if not currently included.

We thank the reviewer for this comment and tried to include the relevant adult literature (see e.g. lines 351-356 and 583-604). In particular, we added a quite detailed discussion about a paper published after our initial submission that performed similar experiments for the adult PAM-DANs Lozada-Perdomo et al., 2025, iScience.

We do not agree, however, that the experiments in Fig. 7 (previously Fig. 6) are redundant. Recent studies in adults found no correlation between the rewarding/punishing effects and the innate valence a given dopaminergic neuron induces (Rohrsen et al., 2021, bioRxiv; Mohammad et al., 2024, PLOS Biol; Lozada-Perdomo et al., 2025, iScience). To our knowledge, no such studies have been carried out in larvae so far. Therefore, we think that it is not only important to test it but that the respective results compared to the results in adults are of relevance for the readership.

The evidence for the role of dopamine (Figure 7) can be bolstered by using other available RNAi lines against TH. A valium20 vector-based shRNA line is recommended. The current evidence is based mainly on non-specific pharmacological intervention with 3IY.

We agree to this caveat and made it transparent now in lines 387-389 and 401-403. We nevertheless chose, for the time being, to not include further experiments to address this point in the current study.

**Recommendations for the authors:**

**Reviewer #1 (Recommendations for the authors):**

Activation of specific or multiple DANs seems to increase naïve odor preference (f1 or TH). Is this due to locomotion defects in TH - how does the odor preference develop over time? Can this increased odor preference explain the safety learning - where they also approach the odor stimulus?

The reviewer is right that in presence of light, we see increased odor preference both innate and after unpaired training – theoretically, that could be the same effect. However, this would not explain why we see the same increased preferences after unpaired learning with all driver strains but increased innate preferences only for some of them. Moreover, when activating TH, we also see increased odor preference in absence of light after unpaired training but not innately. We therefore think that these are independent effects.

We also include a new Fig. 6 providing additional information, including the development of preference over time, and addressing the question whether the modulations in locomotion can explain differences in odor preference.

Locomotion behavior was assessed in 30s light-on periods - was the behavior different from the memory test or naïve preference test which had light on for 3 (or 2.5) minutes - which light intensity was used for the data shown in Figure 5.2? How did the larvae move in the high light concentration/ or with ATR - did they not show memory due to impaired locomotion?

Light intensity for all odour preference and learning experiments with ChR2-XXL was 100 µW/cm^2^ (except Fig. 2 – S2C), i.e. equivalent to the experiments in Fig. 5 – S1 and Fig. 9 – S2 (weak light). We replaced Fig. 5 – S2 with a new expanded Fig. 6 analysing the locomotion of our experiments shown in Fig. 2F, 3F and Fig. 2 – S1F. We show in this figure that the larvae can move relatively normal and that the locomotion effects are weaker than in our experiments with 30s light periods. Unfortunately, we do not have videos available for all experiments and therefore cannot make a similar analysis for Fig. 2 – S2C and D when we used strong light or ATR feeding. The experimenters did not notice impaired locomotion during the experiment and the animals did show normal odour preferences similar to those shown in Fig. 3 – but the preferences were the same after paired and unpaired training, resulting in zero Memory Scores. Therefore, we do not think that the locomotion prevented the memory expression.

In several experiments, even genetic controls seem to show learning with blue light activation. Thus, the light itself seems to activate DANs. The authors should discuss these effects and explain what this could mean for the findings. The light stimulus might not just activate the specific DAN that expresses the optogenetics, but also additionally other DANs which respond to light.

We thank the reviewer to point this out and point out this caveat in lines 159-165.

The authors speculate about the function of potential MB circuits - the DAN-MBON circuit is not well described so far and might be required for the US in test memory recall. A straightforward experiment to investigate the involvement of this circuit in punishment or safety memory recall would be to block dopamine receptors in the MBON.

We very much agree to this suggestion, but believe these experiments are beyond the scope of the current study. We therefore decided to not perform these experiments for the current paper.

**Reviewer #2 (Recommendations for the authors):**

(1) As self-explanatory as the figures are, it would be interesting to also see controls in some of them. For example, in Figure 5, the effect size graph (Figure 5C) clarifies to an extent the difference between control genotypes and the experimental genotypes. It would be nice to see the results of genetic controls in Figures 5A and 5B instead of in Figure 4 - supplement 2.

We originally decided to put the controls into the supplement to prevent the main figures to be overcrowded. We revised this decision upon the reviewer’s comment for Fig. 8 (originally 7). For Fig. 4 and 5 specifically, we decided to keep the current layout because each serves a different purpose: Fig. 4D-L, Fig. 5 – S1 and S2 present the actual data with all genotypes that were made in parallel and therefore can be compared directly. Fig. 4 – S2 aims to visualize the effect of the light by comparing all controls across all experiments, normalized to the same starting value. Fig. 5A and B aim to compare the shape and effect size of activating DANs on top of the effect of the light - therefore, we subtracted the controls in each experiment from the experimental group. We think that adding the controls’ behaviour to Fig. 5A and B would undermine the aim of this figure.

(2) It is a bit unclear why bending and tail velocities were the parameters chosen to compare between groups while in most cases they were of lower relative importance according to Figure 4 - supplement 1. Elaborating on this would strengthen the differences in behavior and also the claims of this study.

We thank the reviewer for the suggestion and tried to make our choice clearer. Please see our answer to the respective part of the public review.

(3) In adults, it has been shown that the same DAN can encode opposing valence depending on whether it was activated before or after odor presentation. Discussing the importance of temporal order of stimulus processing would bolster the results regarding paired and unpaired training in Figure 3.

We thank the reviewer for this very good suggestion – also in larvae, this temporal function has been described. We discuss these observations in relation to our results in lines in 481-494.

**Reviewer #3 (Recommendations for the authors):**

Toshima et al., as stated in the public reviews, have done an admirable job with this manuscript. Below are specific suggestions that could improve the manuscript. It is, of course, up to the authors to decide which ones to attend to.

Treat controls consistently. In Figure 2 and others, parental controls are not pooled, but in Figure 3, for odor preference, controls are pooled.

We agree that the same things should be treated in the same way throughout a study and normally adhere to this principle. We nevertheless made an exception for Fig. 3 only because its goal is to provide a post-hoc analysis across several replications of experiments, some of which included genetic controls, others not (from Fig. 2, Fig. 2-S2 and S3). Due to relatively small effect sizes and high variability in odor preferences, to answer the question of paired and unpaired learning, we need higher sample sizes than each individual experiment provided. We therefore decided to pool all “equivalent” data across all these experiments. We do agree that this is a suboptimal approach but hope the reviewer can understand the rationale behind it. We explained our rationale clearer now (lines 180-183).

I prefer to see all data points in a graph. It is more transparent than the box plots. Also, could you note why the data median is preferable to show over the mean?

Although we in principle agree to the notion that presenting all data points is more transparent, we opted against it as it makes some graphs harder to read in particular with high sample sizes – in some of our figures, we have hundreds of data points per group. We explain our choice, including for using the median, in the method section (lines 835-839).

Please undertake another round of language editing to handle spelling errors, etc. Use consistent British/American English.

We thank the reviewer for their suggestion and tried our best to fix any spelling and grammar errors.

I urge the authors to move beyond the false dichotomy of 'p' value statistics to using the statistical framework of estimation statistics for data analysis. I understand switching from familiar statistical analysis in such a late manuscript stage is very difficult. However, the authors can consider the estimation statistics framework in subsequent studies. https://www.estimationstats.com is a good starting point for biologists to get to know a framework that has been extensively worked on and is arguably a more 'honest' way of analyzing data. Disclaimer: I am not associated with the above website.

We agree that the p-value has problems and are aware of the estimation statistics framework. We had considered applying it here, but we decided against switching to a completely different statistical framework for a research project that was ongoing since several years. However, we are sincerely considering it for our current research projects.
